# Supplementary material for: Structure of the ciliary tip central pair reveals the unique role of the microtubule-seam binding protein SPEF1
Source: bioRxiv. 2024 Dec 2:2024.12.02.626492. Preprint. [Version 1] doi: 10.1101/2024.12.02.626492 (PMC11642885; doi:10.1101/2024.12.02.626492)
Supplement: 1 [file NIHPP2024.12.02.626492V1-supplement-1.pdf]

bioRxiv preprint doi: https://doi.org/10.1101/2024.12.02.626492; this version posted December 4, 2024. The copyright holder for this preprint (which was not certified by peer review) is the author/funder, who has granted bioRxiv a license to display the preprint in perpetuity. It is made available under aCC-BY 4.0 International license.

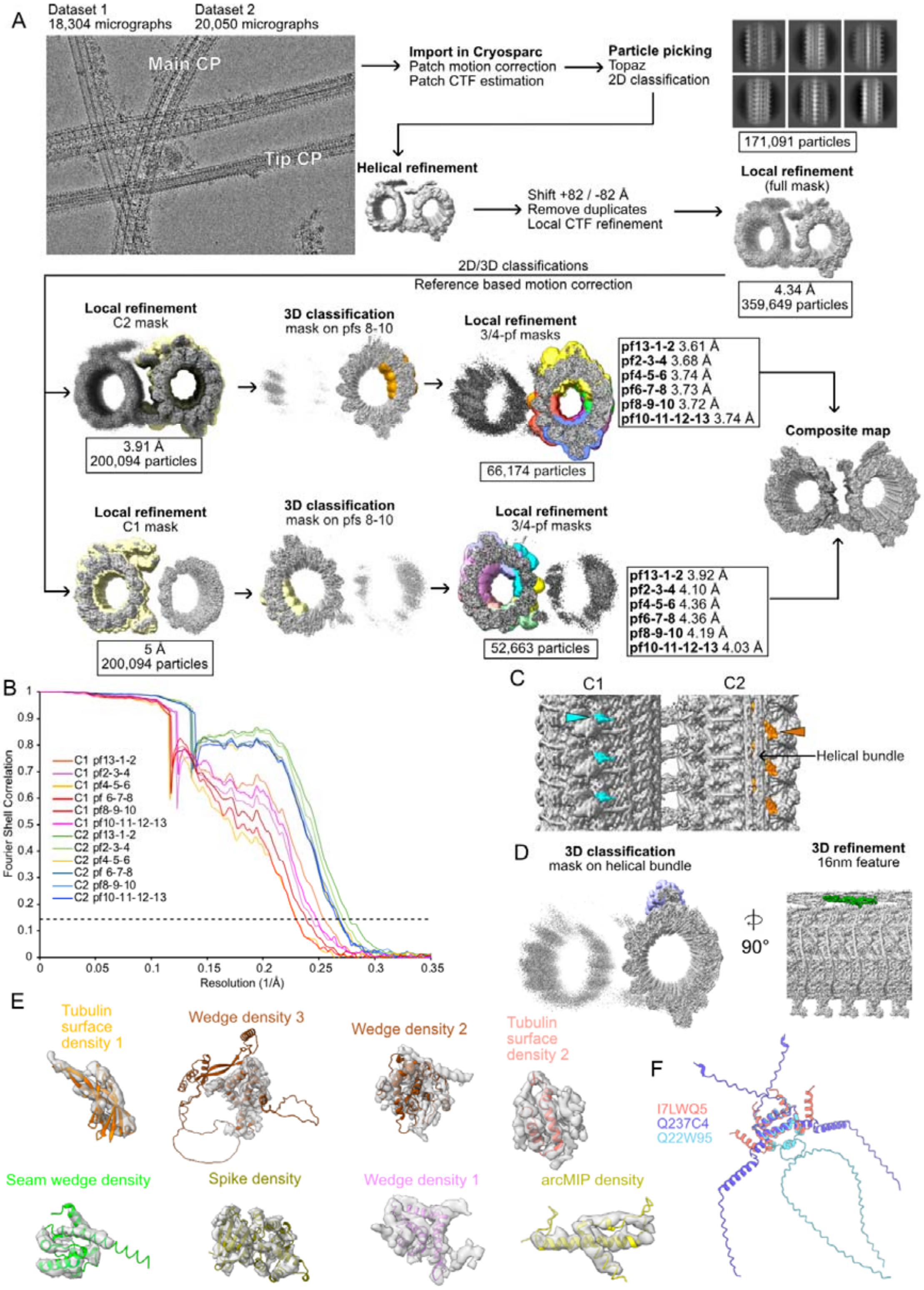

Supplementary Figure 1: *(A) Cryo-EM processing workflow of the tip CP. (B) Fourier shell correlation curves of the local refinements used in the composite map of the tip CP. (C) Densities specific to C1 (blue) and C2 (orange) highlight the asymmetry between the two microtubules. (D) Mask (purple) used for the 3D classification of the helical bundle and the 16 nm feature (green) identified. (E) Densities segmented for use with DomainFit (grey) and best fit of the predicted model for each density (colour). (F) AlphaFold2-predicted dimers of three ciliary DPY-30-containing proteins (UniProt IDs: I7LWQ5, Q237C4, and Q22W95).*

bioRxiv preprint doi: https://doi.org/10.1101/2024.12.02.626492; this version posted December 4, 2024. The copyright holder for this preprint (which was not certified by peer review) is the author/funder, who has granted bioRxiv a license to display the preprint in perpetuity. It is made available under aCC-BY 4.0 International license.

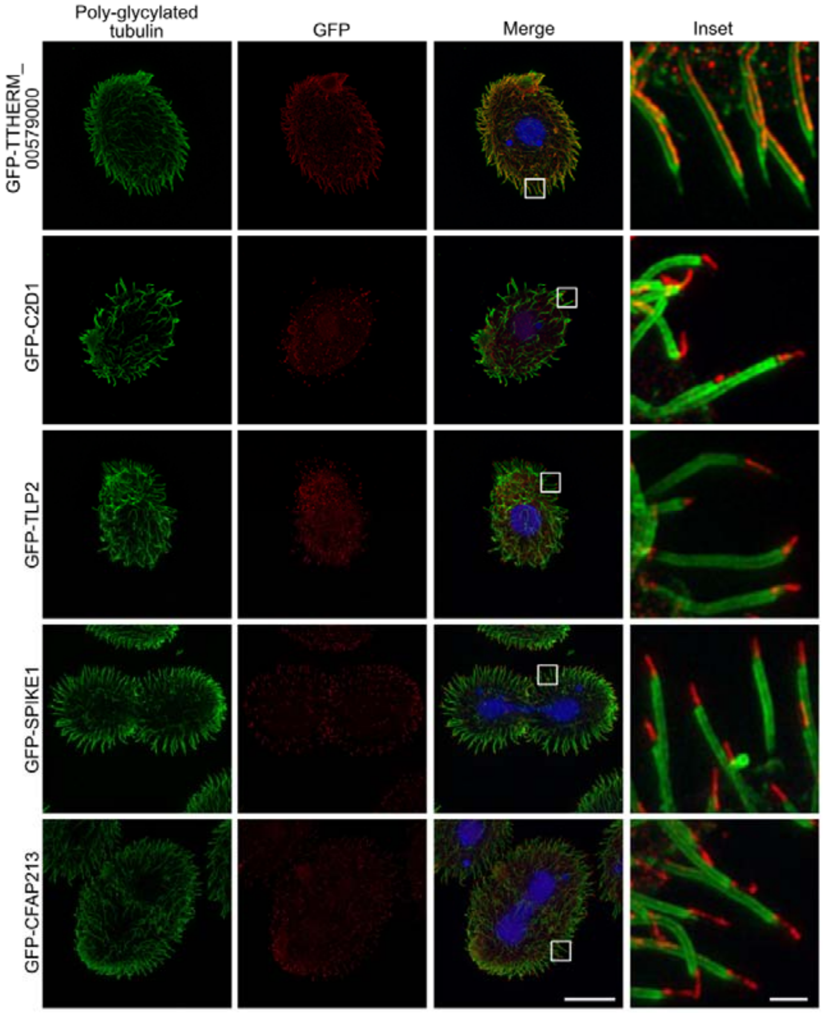

Supplementary Figure 2: *Representative images of the colocalization of polyglycylated tubulin (green) and GFP-tagged TTHERM_00579000, TLP1, C2D1, TLP2, SPIKE1 and CFAP213 (red) and DNA (blue). The last column shows merged images. Scale bar: 20 µm. Insets zoomed in on representative cilia from the cell shown in the merged image. Scale bar: 1.5 µm.*

bioRxiv preprint doi: https://doi.org/10.1101/2024.12.02.626492; this version posted December 4, 2024. The copyright holder for this preprint (which was not certified by peer review) is the author/funder, who has granted bioRxiv a license to display the preprint in perpetuity. It is made available under aCC-BY 4.0 International license.

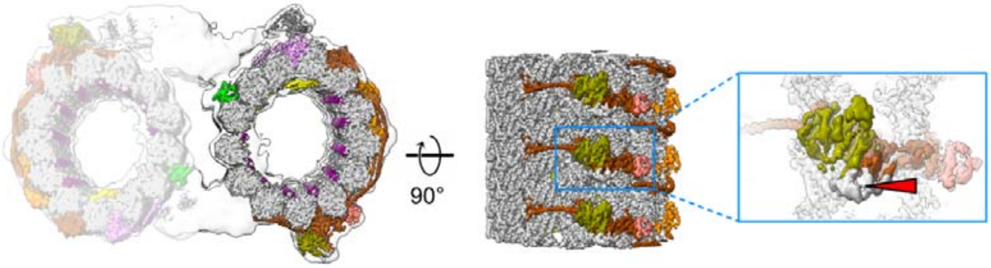

Supplementary Figure 3: *Spike density coloured near the predicted model of SPIKE1 (green) and TLP2 (brown). The red arrowhead points to a density whose protein was not identified (gray).*

bioRxiv preprint doi: https://doi.org/10.1101/2024.12.02.626492; this version posted December 4, 2024. The copyright holder for this preprint (which was not certified by peer review) is the author/funder, who has granted bioRxiv a license to display the preprint in perpetuity. It is made available under aCC-BY 4.0 International license.

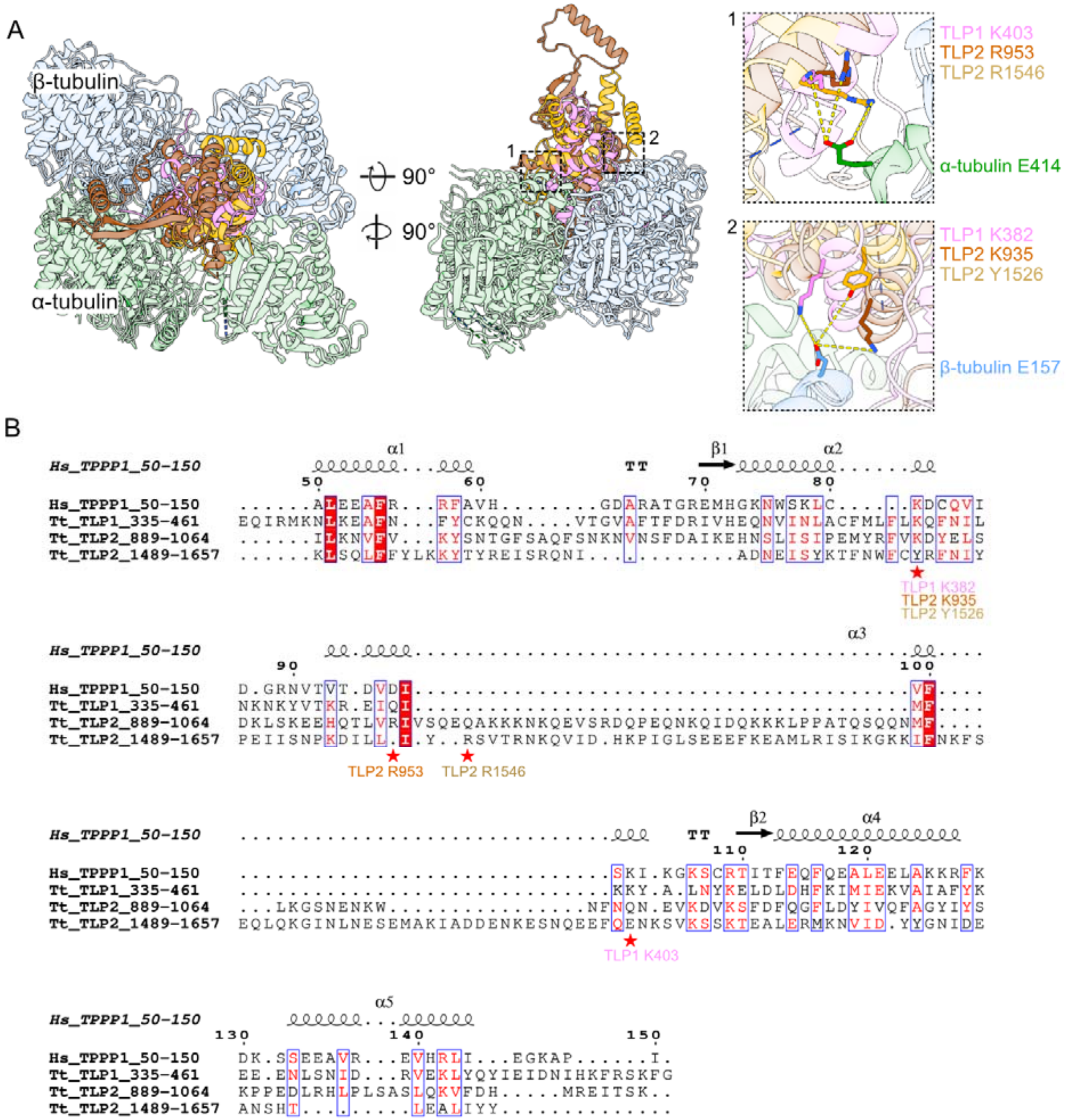

Figure S4: *(A) Models of TPPP-like domains of TLP1 and TLP2 interacting with tubulin. (B) Alignment of the human TPPP1 (UniProtID O94811) and TPPP-like domains of TLP1 and TLP2. The amino acids shown in panel A are indicated by a red star.*

bioRxiv preprint doi: https://doi.org/10.1101/2024.12.02.626492; this version posted December 4, 2024. The copyright holder for this preprint (which was not certified by peer review) is the author/funder, who has granted bioRxiv a license to display the preprint in perpetuity. It is made available under aCC-BY 4.0 International license.

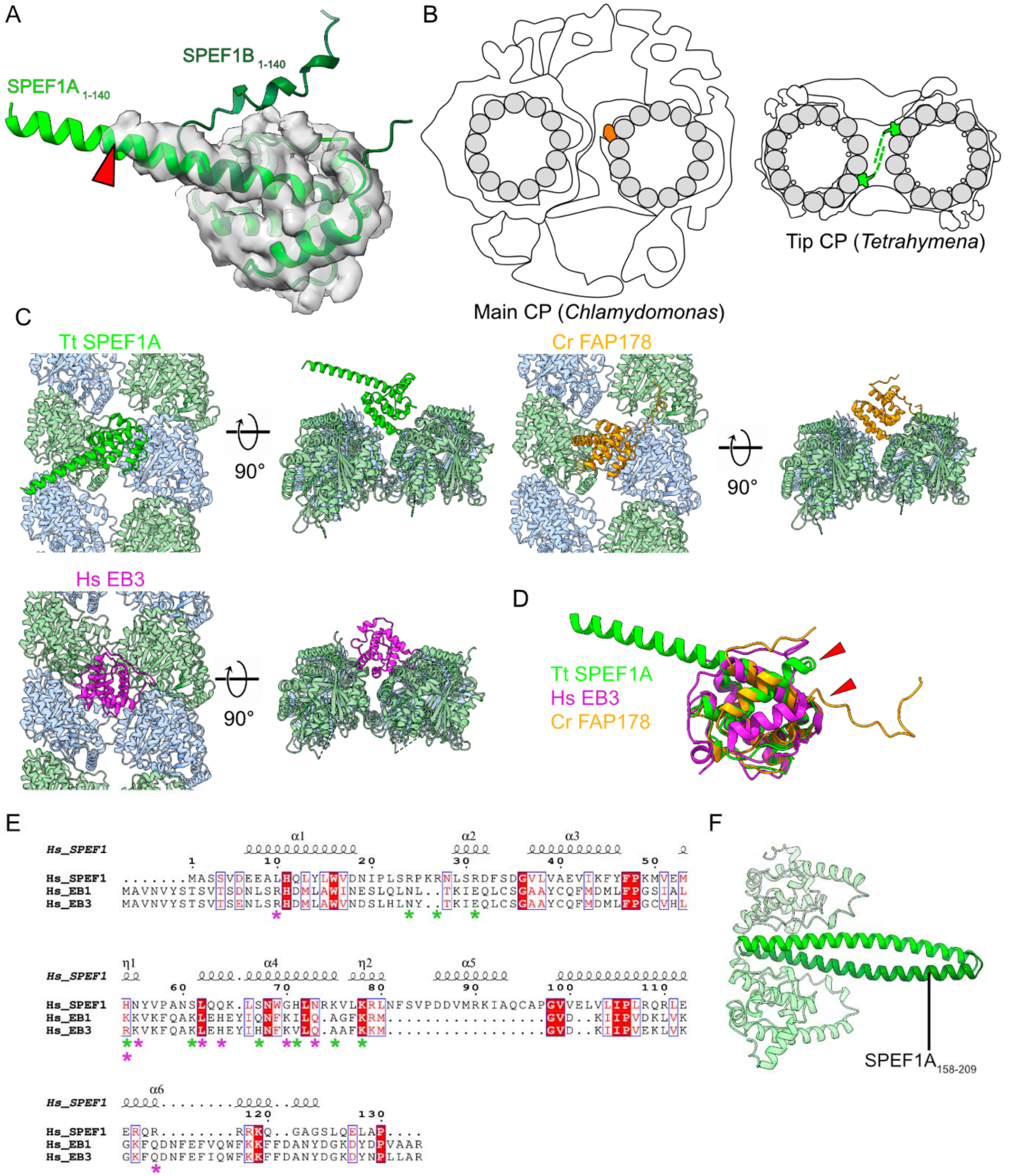

Figure S5: *(A) Overlay of SPEF1A$_{1\text{-}140}$ and SPEF1B$_{1\text{-}140}$. The SPEF1 density from the CP map is in gray. The red arrowhead highlights helix 6 of SPEF1A. (B) Diagrams of the main (left) and tip (right) CPs showing that SPEF1A might dimerize between C1 and C2. CrFAP178 is shown in orange. SPEF1A is shown in green. (C) Models of TtSPEF1A (green), CrFAP178 (orange, EMDB-25361) and HsEB3 (pink, EMDB-6365) interacting with tubulin. (D) Overlay of TtSPEF1A, HsEB3 and CrFAP178. The arrowheads point to a different orientation of helix 1. (E) Alignment of human HsSPEF1$_{1\text{-}130}$ (UniProtID Q9Y4P9), EB3 (UniProtID Q9UPY8) and EB1 (UniProtID Q15691). The amino acids interacting with tubulin are indicated by a purple asterisk for EB3 and a green asterisk for SPEF1A. (F) Predicted Alphafold2 model of a SPEF1A dimer. The helix mediating dimerization (SPEF1A$_{158\text{--}209}$) is highlighted in green.*

bioRxiv preprint doi: https://doi.org/10.1101/2024.12.02.626492; this version posted December 4, 2024. The copyright holder for this preprint (which was not certified by peer review) is the author/funder, who has granted bioRxiv a license to display the preprint in perpetuity. It is made available under aCC-BY 4.0 International license.

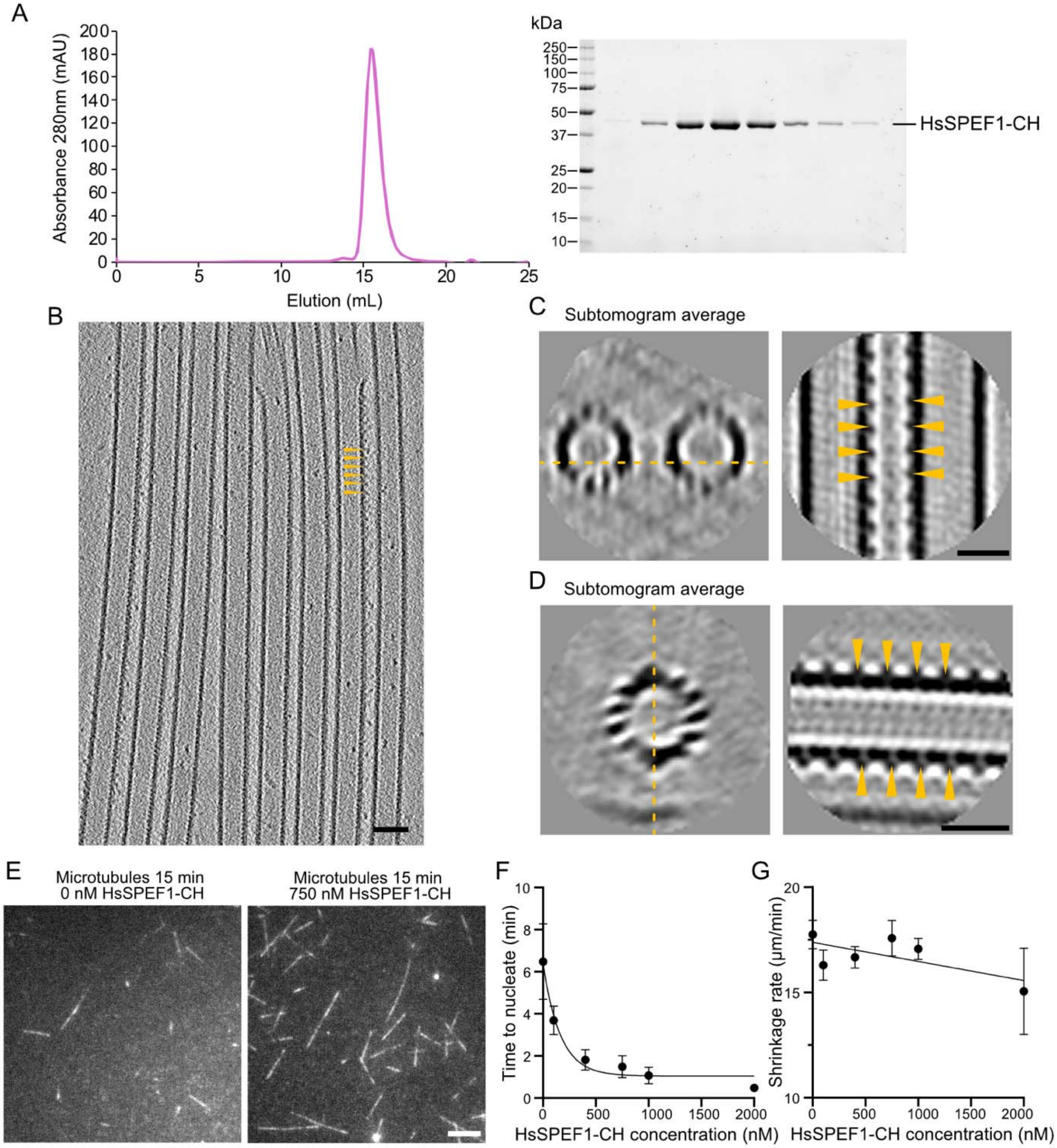

Figure S6: *(A) Curve after SEC and the corresponding gel for HsSPEF1-CH. (B) Example tomogram of microtubules with HsSPEF1-CH. Arrowheads point to extradensity binding to the side of a microtubule with an apparent repeat of 8 nm. Scale bar, 40 nm. (C - D) Subtomogram averages of a microtubule pair shown in Fig. 6F and a single microtubule from another tomogram. Arrowheads point to extra densities that repeat every 8 nm. Scale bar 20 nm. (E) Representative TIRF images of the AF546 channel (microtubules) at t = 15 min with and without 750 nM HsSPEF1-CH. Scale bar, 4 µm. (F) Time for microtubules to nucleate vs the HsSPEF1-CH concentration. (G) Microtubule shrinkage rate vs the HsSPEF1-CH concentration.*
